# A review of the literature on sexual and reproductive health of African migrant and refugee children

**DOI:** 10.1186/s12978-021-01138-3

**Published:** 2021-04-17

**Authors:** Stephen Owusu Kwankye, Solina Richter, Philomina Okeke-Ihejirika, Hayat Gomma, Pamela Obegu, Bukola Salami

**Affiliations:** 1 Present Address: Regional Institute for Population Studies, University of Ghana, Legon, P. O Box LG 96, Accra, Ghana; 2 University of Alberta, Edmonton, Canada; 3 Ahmadu Bello University, Zaria, Nigeria; 4 Heartwood House and Volunteers at Oxfam, Ottawa, Canada

**Keywords:** Child migration, Sexual health, Reproductive health, African migrant, Refugee children

## Abstract

**Background:**

Migration and involuntary displacement of children and young people have recently become common features of many African countries due to widespread poverty, rapid urbanization, joblessness, and instability that motivate them to seek livelihoods away from their places of origin. With limited education and skills, children become vulnerable socioeconomically, thereby exposing themselves to sexual and reproductive health (SRH) risks.

**Methods:**

Against this background, the authors undertook a scoping review of the existing literature between January and June 2019 to highlight current knowledge on SRH of African migrant and refugee children. Twenty-two studies that met the inclusion criteria were reviewed.

**Results:**

The results identified overcrowding and sexual exploitation of children within refugee camps where reproductive health services are often limited and underutilized. They also reveal language barriers as key obstacles towards young migrants’ access to SRH information and services because local languages used to deliver these services are alien to the migrants. Further, cultural practices like genital cutting, which survived migration could have serious reproductive health implications for young migrants. A major gap identified is about SRH risk factors of unaccompanied migrant minors, which have received limited study, and calls for more quantitative and qualitative SRH studies on unaccompanied child migrants. Studies should also focus on the different dimensions of SRH challenges among child migrants differentiated by gender, documented or undocumented, within or across national borders, and within or outside refugee camps to properly inform and situate policies, keeping in mind the economic motive and spatial displacement of children as major considerations.

**Conclusion:**

The conditions that necessitate economic-driven migration of children will continue to exist in sub-Saharan Africa. This will provide fertile grounds for child migration to continue to thrive, with diverse sexual and reproductive health risks among the child migrants. There is need for further quantitative and qualitative research on child migrants’ sexual and reproductive health experiences paying special attention to their differentiation by gender, documented or undocumented, within or across national borders and within or outside refugee camps.

## Plain language summary

Child migration is increasingly becoming a common feature of many African countries as children migrate within and away from their countries of origin in search of better livelihoods due to widespread poverty, instability and rapid urbanisation. Many of the migrating children have little or no education and limited employable skills, thereby exposing themselves to sexual and reproductive health (SRH) risks. This paper is a scoping review of existing literature from 2000 to 2019 to document relevant findings on SRH of African migrant and refugee children.

The review identified migrant children within refugee camps to experience overcrowding and sexual exploitation amidst limited and underutilized reproductive health services. Language barriers are also key obstacles against young migrants’ access to SRH information and services as these services are delivered in local languages unfamiliar to them. Child migrants continue to suffer from cultural practices including genital cutting with serious reproductive health implications. SRH risk factors of unaccompanied migrant minors remain as a gap in the review and, therefore, more SRH studies on unaccompanied child migrants are needed. Attention should also be paid to the different dimensions of SRH challenges among child migrants to reflect gender differences, whether documented or undocumented, within or across national borders, and within or outside refugee camps for better understanding.

In conclusion, child migration in Africa will continue to be economic-driven and will expose the actors to diverse sexual and reproductive health risks. Further in-depth quantitative and qualitative research is required to inform relevant policies on child migrants’ SRH.

## Background

Africa is in a demographic transition that is ushering into many countries a rapidly growing youthful population. At the same time, the economies of many of these countries are wrestling with instability, unemployment, and a large informal sector characterized by low and unsustainable incomes. Thus, many sub-Saharan African youth are growing up in an environment of widespread poverty, poor employment prospects, rapid urbanization, limited educational opportunities, and rapid socio-cultural transformations characterized by weakening social controls and the breakdown of traditional norms [1]. This situation has engendered the quest for many young people, especially those with little or no education, to embark on cross-border or within-border migration. These have exposed many children and young people in the region to sexual and reproductive health challenges with serious implications for their health and general well-being.

Since 1994, following the holding of the International Conference on Population and Development (ICPD), there has been a paradigm shift in the conceptualization of sexual and reproductive health, especially among young people. The ICPD conceived reproductive health as a matter of human rights, meaning that people can have a satisfying and safe sex life and that they have the capability to reproduce and the freedom to decide if, when, and how often to do so [2]. Notwithstanding this declaration, the policy and socio-cultural environment in many African settings does not appear to be supportive of the operationalization of this new understanding in several countries in sub-Saharan Africa.

The exposure to sexual and reproductive health risks and vulnerabilities is higher among child and unmarried female adolescent migrants. According to He et al. [3], for example, the unmarried female migrant is one of the most vulnerable groups recorded in their study. Anecdotal reports suggest that in response to a high level of poverty, unemployment, and low education, some adolescents frequently embark on rural–urban migration within their countries and others engage in cross-border movements, which could fuel their exposure to sexual and reproductive health risks and challenges among them. According to Save the Children [4], boys and girls in transition are seeking to venture into new environments where the social and economic dynamics are not well understood by them, neither are they prepared to navigate, thereby exposing them to vulnerable situations of violence, exploitation and limited access to opportunities. As earlier indicated, this is especially the case among unmarried adolescent females since many of them are unable to negotiate safe and protected sex while unaccompanied away from home.

While some studies have been conducted in countries in Africa to examine the sexual and reproductive health of adolescents and young people, there is no comprehensive compilation of the issues on the sexual and reproductive health vulnerabilities of migrant children that highlights the critical knowledge gaps for further investigation. Against this backdrop, this paper’s main objective is to undertake a comprehensive scoping review to document the various dimensions of research that have been undertaken in this important area to inform further relevant studies based on the gaps it will reveal.

## Methods

We used a scoping review methodology that consists of five stages [5]. In the first stage of the scoping review, we identified research questions suitable for a scoping review. Our scoping review was guided by the following research questions:

i What is known from the existing literature on the sexual and reproductive health (SRH) of African migrant and refugee children?
ii What is the scope, range, and nature of the evidence on the sexual health of African migrant and refugee children?

At the second stage of the scoping review, we searched multiple databases including Medline, Embase, Global Health, Psych Info, Cochrane Library, CINAHL, SocIndex, Child Development and Adolescent Studies, Sociological Abstract, and ProQuest Dissertations & Theses Global. The search was completed by an experienced health science librarian in January 2019. We combined search terms related to child (e.g., child, adolescent, youth, pediatric) with search terms related to migrants (refugee, immigrant, asylum seeker, internally displaced persons, etc.) and  search terms related to Africa (including the names of all African countries). The search yielded 12,720 articles. A total of 6,602 articles remained after duplicates were excluded. All articles were then exported to Covidence, an online platform designed for article selection and data extraction of systematic reviews.

From February 2019 to May 2019, we completed the third stage of our scoping review and article selection. Six research assistants were involved in article selection. Each article was reviewed by two research assistants on Covidence, a web-based software that streamlines the scoping review process. Disagreements between research assistants were resolved by consensus. The inclusion criteria were that the article must be focused on the health of African migrant, refugee and/or internally displaced children age 0 to 18 years. We only included first generation migrants (i.e., the children must have migrated). We excluded literature reviews, systematic reviews, and articles focused on the experience of parents (without data on the health of the child). The research assistants met with the last author multiple times to clarify inclusion and exclusion criteria and provide mentorship. The last author also completed frequent quality checks on the research assistants’ screening on Covidence. At this stage, 1,674 articles met the inclusion criteria based on the review of titles and abstracts. The large volume of articles necessitated us to further narrow down our search. Two research assistants further applied the following inclusion criteria: articles that focused on the reproductive and sexual health of children age 0 to 18 years in sub-Saharan Africa and published between 2000 and 2019. We excluded epidemiological studies where African migrants were only a small variable of a bigger study. For studies where data on children’s health were mixed with adults’ health or where data on Africans were mixed with non-Africans, we only included these studies if the data were disaggregated in the report (so as to enable us to analyze the data on African children’s health only). In total, 22 articles met our inclusion criteria and are included in this review.

The fourth stage is data extraction. We extracted the following information from all 22 articles: author name, title, year of publication, research questions or objectives, methodology, theoretical framework, method (sampling, sample size), age of children, data source (parent versus child versus health professional), period of data collection, country of origin or region, destination country or region, summary of findings, and summary of implications (See Appendix Table 1 for some of the information extracted from the articles).

The final stage involved collating, summarizing, and reporting the results. We identified prevailing categories within our data and summarized the articles within those categories.

## Results

The studies that were reviewed highlighted issues relative to Research Question (i) on the SRH of migrant and refugee children from sub-Saharan Africa on five main themes. These were the following: sexual vulnerabilities/exploitation of immigrant children (n = 9), SRH education/communication (n = 4), child protection and resettlement services (n = 3), the prevalence of genital mutilation (n = 3), and adolescent sexual behavior (n = 3).

Concerning Research Question (ii), the 22 studies [6–27] that were included in the review were made up of 13 qualitative, seven quantitative and two mixed methods studies). The sample size ranged from six (6) to 330 in the qualitative studies and from 47 to 2,344 in the quantitative studies. In contrast, the mixed methods studies had a sample size ranging between 53 and 919. Seventeen of the articles reviewed did not specify a theoretical framework, two used a social-ecological framework, one used a Heise model framework, one of them used a youth resilience framework, and another one used an ecological framework to understand violence. The data sources included children (8), parents/caretakers and adolescents (8), parents only (4), and health professionals (2). With regard to the country of study, the review documented the following countries: Democratic Republic of Congo and Ethiopia (n = 1), Rwanda (n = 3), England (n = 2), Ethiopia (n = 2), Uganda (n = 2), Nigeria (n = 2) and one study from each of the following countries: United States of America, Switzerland, Uganda and DRC, Canada, Spain, South Africa, Portugal, Sweden, Zimbabwe with another on Africa in general. Participants originally migrated from the following countries: Democratic Republic of Congo, The Gambia, Senegal, Equatorial Guinea, Ghana, Mali, Nigeria, Somalia, Uganda, Eritrea, Ethiopia, Burundi, Kenya, Rwanda, South Africa, Tanzania, Zambia, and Zimbabwe.

### Sexual vulnerabilities and exploitation

The review identified factors that accounted for migrant children’s vulnerability to sexual maltreatment and eventual exploitation especially among those who lived in refugee/displacement camps. These factors included overcrowding in camps, insecurity, and the “miserable life” in camp layouts [9, 12]. It gave rise to more forms of sexual exploitation such as unwanted physical touching, commercial sex, girl trafficking, and rape, occurring more than once among boys and girls. Other vulnerabilities such as economic hardship, inability to pay school fees, or the desire for independence equally accounted for non-refugee campers’ involvement in sex work which led to further migration to maximize income [10]. When asked in a study to describe their experience during adolescence, respondents indicated that it was a time of both vulnerability and opportunity during which they developed some survival skills [10]. The high prevalence of sexual violation among African migrant children [8] has negative effects on their sexual and reproductive health. There is, therefore, some evidence from the studies reviewed to suggest situations of vulnerabilities that confront these child migrants as well as opportunities they strive to take advantage of.

### Reproductive health education

Investigations into awareness and open communication about adolescent reproductive health followed as the second most explored theme. Some of the studies showed that it was difficult for parents and guardians of African immigrant children, especially girls, to educate or have open communications about pubertal development [22] mostly due to widespread myths and taboos about sex in this region of the world. This gap in SRH communication alongside the fear of stigma, was also reported in studies that explored the disclosure of the HIV-positive status of immigrant parents to their children [7]. For some immigrants, language barrier hindered, for example, access to HIV preventive practices or options for contraception since health education at health centres was delivered in the local language that was alien to the migrant children [13]. Other parents admitted that their African cultural background does not encourage open discussions about sexual and reproductive health issues with adolescent children. They perceived Americans as being permissive of sexual behaviours, open in discussing sexual behaviours, and not ending conversations about sex initiated by their daughters [6]. Adolescents felt uneasy discussing sexual issues with their parents, leaving their parents and themselves less informed in this regard. Therefore, culture plays a role in the perspectives and practices among the population relative to SRH. Quite clearly, open communication about SRH with immigrant children is an essential part of their SRH development and yet, many of them are denied this opportunity.

### Prevalence of genital mutilation

Circumcision, or the removal of part of the external genitalia, is reported in some of the studies to be prevalent among immigrant children, especially girls. This prevalence is explained to be greatly accounted for by the culture and practices of some African immigrant parents who view it as an obligatory social and traditional norm [17]. According to Vogt et al. [25], an increased prevalence of female genital mutilation is practiced for the same reasons (as a cultural practice) and is further reported to be supported by the immigrants. In situations where children were not yet circumcised, the intention of parents to cut them in the future was strongly expressed [15]. Immigrant male children were also non-therapeutically circumcised by non-medical professionals, usually older women, as a way of sustaining a cultural practice of the places of origin of these immigrants.

### Adolescent sexual behaviour

Puberty marks the beginning of physical changes in adolescents, but the sexual and reproductive preferences are shaped by behaviours that follow. The studies reveal that adolescents recognized the pubertal changes as signs of early adulthood; they also point to cultural patterns that exist that account for inequitable relations between boys and girls [19]. These culturally fostered inequities greatly furthered the practice of early marriage among immigrant female children [19, 21]. Immigrant children are exposed to their culture from home and to the culture of the host country. The effect of this juxtaposition is seen in their sexual behaviour, which exposes them to both health and reproductive-related risks.

### Child protection and resettlement services

Immigrant children who lived in refugee and/or displacement camps were open about their journey of resettling while showing gratitude for the chance to start afresh. They generally expressed the importance of social support, social connectedness, and meaningful social relationships, or sometimes the lack thereof, as important in enabling asylum-seeking and refugee groups to resettle or re-integrate in their home countries [24]. While there were mixed reactions about the habitability of the camps, the issue of underutilization of SRH services in the refugee camps was very common. A situation described as the social consequences of reporting or disclosing abuse by utilizing a formal reporting/referral process emerged as adolescents associated their low utilization of such a key SRH service to the fear of retribution, shame, embarrassment, and social rejection [9]. Total protection and provision may be impossible, as acknowledged by service providers [26], owing to the scarcity of resources and loopholes in reproductive health service provision systems within the refugee camps. Adolescent girls seemed to rely on their interpersonal and communal relations as a source of protection [23]. An obligation society has towards children is protection. Unfortunately, this often eludes them, going by the cries of the unheard voices of unaccompanied or accompanied immigrant children who live through the realities, especially the refugees.

## Discussion

The review has presented issues that when carefully examined could aid in addressing the sexual and reproductive health challenges of migrant and refugee children in Africa, especially when viewed from the gaps that were identified regarding SRH communication between immigrant parents and their children [7]. Also important is the issue of refugee camps in Africa, which are characterized by overcrowding, which resulted from the unpreparedness of most host countries to receive displaced persons in times of civil and political destabilization in the countries of origin. Situations of overcrowding often present fertile grounds for the sexual exploitation of children and young girls. In Ghana, for example, young migrant girls who are compelled to sleep in front of stores and shops at market centres and at the city centres tend to be exposed to sexual exploitation including rape [28]. This is especially when boys and girls congregate together at night in open spaces.

Another source of the vulnerability of migrant and refugee children to sexual and reproductive health challenges, which was evident in the review, was the language barrier that undermines their access to SRH services. In many sub-Saharan African countries, adolescents and young people face socio-cultural barriers in their quest to access SRH information and services. A study in Tanzania reported that both community members and service providers hold the view that it is not appropriate for girls age 10–18 years to access sexual and reproductive health services, especially family planning [29]. Accessing SRH services among young people attracts stigma and discrimination in the community where this study was conducted. In a focus group discussion, one adult male was reported to have said, for example, “I do not think it’s right for young people to use family planning methods since it will affect their reproductive health system and unable them to get children” [29]. This barrier in accessing SRH services is also underscored by Save the Children [4] in their observation that both adolescent boys and girls face challenges in accessing services in their new locations while in transition and are confronted with limited livelihood opportunities on arrival in their new destination areas outside their home areas of origin.

There are documented perceptions that contraceptives are for adult married people and not for unmarried adolescents and, yet many unmarried adolescents are sexually active. This is supported by a study on health-care providers’ attitudes toward the provision of contraceptives for unmarried adolescents in Ibadan, Nigeria [30]. In the Nigerian study, it was reported that 57.5% of the respondents were of the view that providing contraceptives to unmarried adolescents would promote sexual promiscuity and 42.7% said unmarried adolescents should not be provided with contraceptives because the Nigerian culture does not support premarital sex [30]. This means that if a large proportion of family planning service providers at the destination communities of child migrants and refugee children hold such beliefs, it could be a big barrier for adolescents who desire to access SRH information and services. This could result in unplanned or unwanted pregnancies or sexually transmitted infections among the sexually active ones that may resort to unprotected sexual activities in the absence of appropriate services that could get them protected against these unwanted reproductive health outcomes.

For the migrant and refugee children at the destination country, the language barrier was noted in some of the studies as a critical issue for accessing reproductive health information as well as services. The persistence of such language barriers may predispose many of them to be alienated and excluded from any window of opportunity in already-unfriendly environments that are insensitive to their need for sexual and reproductive health services.

It was also clear from the review that access to reproductive health services among the migrant and refugee children is hampered by the myths associated with sex in their communities of origin, which do not empower young people to seek information and services. It has also been argued that when people migrate, they are exposed to behaviours and norms that tend to be different from those of their place of origin [31]. Thus, it is possible that as the migrant children stay longer in the destination country, they may be influenced by the prevailing sexual behaviour norms in the destination country to negotiate their way out of the myths surrounding sex at the places of origin. As a result, a young migrant’s sexual behaviour may not necessarily be shaped by the myths at the country of origin but instead by the degree of freedom they may experience in their sexual and emotional life in addition to the general level of permissiveness in the host society. Language barriers also tend to constitute a greater challenge among newly arriving child migrants compared to other persons who have stayed in the countries of destination for a much longer time. The latter may already have acquainted themselves with the local languages of their host communities and hence, are better able to navigate sexual behaviours that are different from those left behind at the country of origin. It is also important to stress that in many African countries, many borders often separate people of similar cultures and language groups between countries along common borders. As a result, depending on how far the child migrant moves away from the borders separating the origin and destination countries, language may not constitute a barrier since in many instances, the people along either side of the borders may speak the same or similar languages that may be understood by both of them. Therefore, it is not in all cases that language could be a stumbling block in the way of child migrants who seek SRH information and services in the destination country.

Another critical issue that emerged from the review is female genital cutting that was situated within the cultural context. While this is true, in many countries in Africa such as Nigeria female genital cutting has been outlawed and yet continues to be practised, perhaps due to its link to tradition and culture which many societies still hold, even away from their country of origin. For example, according to an article published in the International Business Times, many girls in Nigeria are still subjected to female genital mutilation despite laws banning the practice [32]. This is interesting because there are anecdotal reports in Ghana that point to children who may be forced to migrate to avoid being subjected to genital cutting against their will. However, in situations where societies are displaced and people are forced to relocate by moving to host countries where female genital cutting is not performed, they still practise genital cutting. This is explained by a clear indication that migrant and refugee parents tend to have a strong bond with the socio-cultural belief systems and practices from their countries of origin. It should, however, be noted that in some cases the migration may be just across a border that simply cuts across one ethnic group that straddles two or more countries. In Africa, such situations abound across borders separating different countries. Therefore, the continuation of such cultural practices after migration into another country may not be a strange development since the socio-cultural environment at the destination may not necessarily be different from that at the origin. It is also the case that in many countries in Africa, female genital cutting has been criminalised although it may be practised under cover in some traditional societies. It may, therefore, be an exaggeration of the situation of female genital cutting as reported in some of the studies reviewed among the immigrant children. Caution should be exercised about the evidence of female genital mutilation reported in some of the studies presented in this review.

Again, some of the studies pointed to gender differentiation relative to SRH in terms of the culturally fostered inequalities between boys and girls. Until quite recently, in many sub-Saharan African countries, the girl child was disadvantaged concerning education and so while the boy child often was taken to school, his girl child counterpart was frequently left behind and cut off from schooling especially after post-primary school. Although much of this is changing, there is still some work to be done to bridge the gender disparity gap, which is confirmed by the early marriages some of the migrant girl children experienced as was reported in some of the studies. Thus, the girl child is more exposed to sexual and reproductive health vulnerabilities due to her relatively lower education and little or no possession of employable skills in addition to her inability to negotiate safe sexual activity because of weak economic strength to fend for herself without depending on male sexual partners, in many instances.

### Gaps

While migration and displacement of children from sub-Saharan Africa are the result of several reasons that range from economic hardship to the desire to be independent [10, 16], there is still a need to conduct studies on SRH risk factors of unaccompanied minors as this was understudied with only one study focusing on unaccompanied migrant minors [14]. There is also a need to conduct follow-up studies to investigate the association between the exposure of these unaccompanied minors to the risk factors observed and the development of adverse health outcomes. Although 13 out of the 22 studies reviewed were qualitative studies, in comparison to the seven that used quantitative methods, there is a need for more qualitative research that will further elucidate the reproductive and sexual health needs and concerns of migrant and refugee children from sub-Saharan Africa beyond surveys and predetermined sets of interview questions.

Perhaps, the conduct of more mixed methods-driven studies could assist in providing more insight into the underlying explanations of sexual and reproductive health vulnerabilities among young migrant children in Africa. The qualitative aspects of such studies could lend further in-depth explanation for some of the quantitative analytical results. Such studies have the advantage of giving a holistic overview of the situation in different country settings either as destination or origin of child migrants.

Most of the studies reviewed do not distinguish between the situations of accompanied and unaccompanied children and their exposure to sexual and reproductive health risks after their migration. Most of them lump them together and it is, therefore, not possible to discuss possible differences between the two groups of migrant children. This gap in the review requires targeted research to aid our enhanced understanding of the possible differences between the two groups of child migrants.

## Conclusion

In most of Africa, child migration is mainly driven by economic motives. There is always an element of economic motive that underlies child migration decision making. On the other hand, some are forced to migrate due to internal displacement circumstances resulting from natural disasters and civil and political instability. As long as the conditions that necessitate economic-driven migration of children continue to exist in sub-Saharan Africa, child migration will continue to thrive with its attendant exposure of the actors to sexual and reproductive health risks. While non-migrant children and adolescents at the country of origin do experience similar sexual and reproductive health challenges just like their migrant counterparts, the challenges are higher among the migrants.

However, migrant social networks provide safety nets to support many of the migrant children to address their reproductive health vulnerabilities at their destination countries or places of residence through the adoption of certain coping strategies. At the refugee camps, the establishment of the camps should factor sexual and reproductive health information and service provision right from the inception of the camps to overcome the underutilization of these services. More quantitative and qualitative studies could provide a more in-depth understanding of the different dimensions of sexual and reproductive health challenges among child migrants differentiated by gender, documented or undocumented, within or across national borders and within or outside refugee camps. This is important to inform different policy decisions targeted at the different actors and situations involved in child migration, particularly within sub-Saharan Africa where the economic motive for the migration of children is always a major consideration.

## Data Availability

There were no primary data collected for the paper. However, details of the review have been summarised in a table and included in the paper.

## Appendix

See Table 1.

**Table 1:** Summary of information from reviewed articles

Author name and year of publication	Title	Summary of findings
Agbemenu, K., M. Hannan., J. Kitutu., M. A. Terry., and W. Doswell. 2017. [6]	“Sex will make your fingers grow thin and then you die”: The interplay of culture, myths, and taboos on African immigrant mothers’ perceptions of reproductive health education with their daughters aged 10–14 years	(1) Mothers in this study were from cultures that did not condone premarital sex—or boy-girl relationships in adolescence. Although it might appear contradictory, as per conversation with the mothers, an element of protectiveness was to be open with their daughters. Because of the perceived permissiveness of sexual behaviours in America, mothers articulated a desire to ward off negative consequences of sexual activity by providing reproductive health education early when daughters were at a younger age and not limit conversations initiated by daughters (2) Myths and taboos about sexual issues are widespread in Africa and are propagated to control sexual behaviour, especially that of unmarried people, particularly women
Åsander, A., A. Björkman., E. Belfrage., and E. Faxelid. 2009. [7]	HIV-infected African parents living in Stockholm, Sweden: Disclosure and planning for their children’s future	In Sweden, the majority of HIV-infected parents are of African origin and despite the introduction of ARV medication, there has been no tendency for disclosures to children to increase. The fear of a double stigma—that of being both an immigrant and HIV infected—is likely the main barrier to the disclosure of HIV infection. HIV-infected immigrants’ custody arrangements for their children remain critical because of their lack of immediate access to family members
Stark, L., K. Asghar., G. Yu., C. Bora., A. A. Baysa., and K. L. Falb. 2017. [8]	Prevalence and associated risk factors of violence against conflict–affected female adolescents: a multi–country, cross–sectional study	(1) Approximately half of the adolescent girls in the sample (54.4% in DRC, 50.5% in Ethiopia) reported experiencing at least one form of violence victimization in the previous 12 months. (2) The prevalence of physical, emotional, and/or sexual violence victimization among adolescent girls (51.62%) is similar to the regional prevalence of the past year’s violence estimated from census data of girls and boys aged 2–14 (50%) and 15–17 (51%) in Africa. Females are at the highest risk for violence during adolescence and persistent gaps in knowledge of violence victimization have, to date, limited the humanitarian community’s ability to appropriately respond to and prevent violence against adolescent girls in these contexts
Bermudez, L., L. Parks., S. R. Meyer., L. Muhorakeye., and L. Stark. 2018. [9]	Safety, trust, and disclosure: A qualitative examination of violence against refugee adolescents in Kiziba Camp, Rwanda	(1) Adolescent respondents noted food insecurity and unemployment as factors that hindered their sense of safety and security. (2) Lack of sufficient housing (overcrowding) were routine protection risks faced in the camp, which could lead to sexual abuse. (3) The fear of punishment or a general feeling that caregivers dismiss adolescent claims of abuse made adolescents less willing to disclose violence or abuse to their caregivers and seek support. (4) The social consequences of disclosing abuse using the formal reporting and referral process are the reason for low utilization of SRH services in refugee camps
Busza, J., S. Mtetwa., P. Chirawu., and F. Cowan. 2014. [10]	Triple jeopardy: Adolescent experiences of sex work and migration in Zimbabwe	Mobility was routine throughout childhood due to family instability and economic hardship. The determinants of mobility, e.g. inability to pay school fees or a desire for independence from difficult circumstances, also catalyzed entry into sex work, which then led to further migration to maximize income. Respondents described their adolescence as a time of both vulnerability and opportunity during which they developed survival skills
Gaspar de Matos, M., T. Gaspar., B. Simons-Morton., B. Reis., and L. Ramiro. 2008. [11]	Communication and information about “safer sex”: intervention issues within communities of African migrants living in poorer neighborhoods in Portugal	(1) African adolescents tend to begin sexual life early, use condoms infrequently, and have more difficulties in talking with parents about sex. (2) There are cultural-specific educational needs of African migrant adolescents. Boys and girls agreed that condom use decision making is the preserve of the male, and girls viewed even unprotected sex better than not being cared for. Some girls indicated that getting pregnant young was normative. (3) Both parents and adolescents expressed inhibition and lack of interest in talking to each other about sex and related issues. Both adolescents and parents think parents were poorly informed about sex-related issues to allow open discussion. Sexuality and HIV were considered taboo themes for parents and adolescents
Iyakaremye, I., and C. Mukagatare. 2016. [12]	Forced migration and sexual abuse: experience of Congolese adolescent girls in Kigeme refugee camp, Rwanda	Rape, unwanted physical touching, sexual exploitation, commercial sex, early marriage, and girl trafficking are the main forms of sexual abuse and exploitation. These are facilitated by the miserable life in the camp, shortcomings in the camp layout and security system, and adolescent developmental stage. They negatively impact girls’ reproductive health, social integration, and mental health
Kunnuji, M., S. Adejoh., U. Esiet., and A. A. Esiet. 2013. [13]	Migration status, reproductive health knowledge and sexual behaviour among female out-of-school adolescents in Iwaya community, Lagos, Nigeria	(1) Migrants are less knowledgeable about HIV and AIDS. Reasons for the observed differentials in knowledge of HIV/AIDS are found in the social exclusion theory which recognizes migrant status as a basis for social exclusion. Migrants may be disadvantaged because they cannot speak local languages well. It is not uncommon, for example, to find situations where information about HIV and AIDS is made available to people in local languages to ensure that most comprehend the messages. (2) Generally, awareness of contraceptive methods was very low among the participants. The methods most known by the participants were male condom and pills which were known to 29 and 10% of the participants respectively
Lay, M., and I. Papadopoulos. 2009. [14]	Sexual maltreatment of unaccompanied asylum-seeking minors from the Horn of Africa: A mixed method study focusing on vulnerability and prevention	A range of sexual maltreatment, including sexual harassment and rape was reported. Three-quarters of the participants experienced more than one incident. Most initial incidents happened in the first 12 months of their arrival in the UK. Two perpetrators were female carers. Many participants reported being groomed and sexually maltreated by people from their own country. Many described being seriously sexually maltreated, particularly by groups of young males living in the same accommodation or nearby, some reportedly also asylum seekers. Participants that had been warned of the dangers of sexual maltreatment were more likely to both disclose and to seek professional help
Macipe-Costa, R., N. García-Sanchez., L. A. Gimeno-Feliu., B. Navarra-Vicente., J. M. Jiménez-Hereza., I. Moneo-Hernández and P. Lobera-Navaz. 2014. [15]	Non-therapeutic male circumcision performed on immigrant children from Africa in Spain	There is non-therapeutic male circumcision among the African immigrant population in Spain. Half (49.1%) of the families who had the circumcision performed in Europe did so at home. The individuals who performed these circumcisions were not health professionals in 84.6% of the cases. Almost all the parents of uncircumcised children had the intention of circumcising them in the future, mostly in their countries of origin
Mberu, B., and M. J. White. 2011. [16]	Internal migration and health: Premarital sexual initiation in Nigeria	(1) Urban–rural migration is a route of transmission as urban migrants returning to rural areas infect their partners with HIV/AIDS. (2) Migrants with previous exposure to urban environments have an increased likelihood of high-risk sexual behaviour in rural areas. (3) Living arrangements and livelihood opportunities that give young people the most physical and financial independence from their parents and relatives expose them to a significant risk of premarital sexual initiation
Mitike, G., and W. Deressa. 2009. [17]	Prevalence and associated factors of female genital mutilation among Somali refugees in eastern Ethiopia: a cross-sectional study	The practice of Female Genital Cutting (FGC) in many societies is considered an obligatory social and traditional norm mainly to maintain virginity and sexual chastity and to reduce and control female sexuality
Odger, A., S. Frohlick, and R. Lorway. 2019. [18]	Re-assembling “risky” subjects: African migrant youth in Winnipeg, Canada	The study identified three trajectories of sexual health messaging in which African immigrant and refugee young women encountered different formations of messaging while living in Winnipeg and travelling between home, school, and university: trajectories of ubiquity, of events and incidents, and of racialized bodies. (1) The first trajectory traced how multiple factors—particularly immigration— shaped how and where interlocutors understood the concept of sexual health, and the visibility of specific issues (i.e., HIV/AIDS, pregnancy, STIs) was largely dependent on the context, emphasizing the everyday nature of sexual health messaging. (2) The second builds upon this ubiquity of sexual health messages by focusing on key moments in their lives that illustrate how risk is actively made in concrete events and incidents along trajectories. (3) The last examines how sexual health messages targeting particular bodies are negotiated and reworked through the experiences and perspectives of young newcomers seeking belonging in places of settlement always against the way they are racialized in the city of Winnipeg
Ortiz-Echevarria, L., M. Greeley., T. Bawoke., L. Zimmerman., C. Robinson., and J. Schlecht. 2017. [19]	Understanding the unique experiences, perspectives and sexual and reproductive health needs of very young adolescents: Somali refugees in Ethiopia	(1) Adolescents demonstrated relatively high comfort with body change during puberty. Both male and female very young adolescents (VYA) identified menstruation, hair growth, breast development, and voice change as signs of becoming an adult. Participants shared that they learned sexual reproductive health information primarily from parents, but also from siblings, peers, and religious leaders. (2) Early marriage was a widely expressed concern among VYAs, particularly among young girls. In general, boys have more opportunities to pursue their education and personal development than girls of similar ages due to community expectations around marriage. (3) While there is wide recognition that male and female roles have been changing since displacement, cultural patterns persist that reinforce inequitable relations between boys and girls in early adolescence
Patel, S., H. Muyinda., N. K. Sewankambo., G. Oyat., S. Atim., and P. M. Spittal, 2012. [20]	In the face of war: examining sexual vulnerabilities of Acholi adolescent girls living in displacement camps in conflict-affected Northern Uganda	(1) The erosion of traditional mentoring systems and cultural norms that previously governed girls’ sexual behaviour and provided cultural cohesion and guidance in supporting and protecting young girls from risky behaviours; these systems and norms have largely been eroded by war-induced displacement. (2) Combined with the collapse of livelihoods, being left in camps unsupervised and idle during the day, commuting within camp perimeters at night away from the family hut to sleep in more central locations due to decreased privacy, and heightened security issues have a direct influence on the girls’ vulnerability in refugee camps
Schlecht, J., E. Rowley., and J. Babirye. 2013. [21]	Early relationships and marriage in conflict and post-conflict settings: vulnerability of youth in Uganda	Marriage was defined as any union accepted by the community to be “marriage”, excluding courtship and dating. Participants distinguished between formalized marriage (which included traditional or religious ceremonies and the exchange of a bride price) and informal marriages acknowledged by the community over time. This research did not explicitly explore forced marriage. The increased practice of early relationships among adolescents, combined with the expansion of informal marriages, was a theme that was consistent throughout interviews in both focus group discussion and in-depth interview settings
Sommer, M., M. Muñoz-Laboy., A. Williams., Y. Mayevskaya., K. Falb, G. Abdella., and L. Stark. 2018. [22]	How narratives of fear shape girls’ participation in community life in two conflict-affected populations	It is difficult for caregivers to talk to their daughters about pubertal development and sex and to have practical discussions on preventing sexual violence. In the absence of having adequate communication tools for such topics, caregivers described obtaining cues about their daughter´s physical and social maturation-related experiences by observing changes in their bodies
Sommer, M., M. Munoz-Laboy., W. E. Salamea, J. Arp., K. L. Falb., N. Rudahindwa., and L. Stark. 2018. [23]	How gender norms are reinforced through violence against adolescent girls in two conflict-affected populations	The study findings provide insights into primarily female attitudes towards experiences of, rationales for, and approaches for risk reduction and response to gender-based violence occurring at the community and interpersonal levels. Overall, female perceptions of safety for adolescent girls were socially produced through girls’ everyday interpersonal interactions, including community and family perceptions of who is responsible for safety and local perceptions of appropriate (or socially inappropriate) responses to violence
Thommessen, S., P. Corcoran., and B. K. Todd. 2017. [24]	Voices rarely heard: Personal construct assessments of Sub-Saharan unaccompanied asylum-seeking and refugee youth in England	Social support, social connectedness, and meaningful social relationships, or sometimes the lack thereof, are important in enabling asylum seeking and refugee groups to resettle in their home countries
Vogt, S., C. Efferson., and E. Fehr. 2017. [25]	The risk of female genital cutting in Europe: Comparing immigrant attitudes toward uncut girls with attitudes in a practicing country	Sudanese immigrants in Switzerland have systematically more positive attitudes toward uncut girls than non-migrants and selective migration from Sudan likely contributes to this difference; migrants vary in their support for female genital cutting
Warria, A. 2018. [26]	Challenges in assistance provision to child victims of transnational trafficking in South Africa	In as much as it is necessary to acknowledge that trafficked children need to grow up in a safe and secure environment, it is also necessary to address the service provider’s concern that provision for total security might not be possible. At the same time, mechanisms put in place to achieve the children’s safety need not downplay the risks that the trafficker poses, nor take away the child’s carefree and fun experiences of childhood. Culturally responsive care becomes a highly important practise method for a child welfare practitioner when engaging with transnational trafficked children from diverse socio-cultural, linguistic, economic, and ethnic backgrounds. This is because trafficked children’s socio-cultural backgrounds will most likely influence the way they perceive the trafficking situation, their expectations for assistance provision, and how they respond to social work interventions. Thus, part of the practitioner’s role during needs assessment is to gain an understanding of the child’s background and cultural factors that could be affiliated to or that might have contributed to the child being trafficked and those that can be applied during therapy and integration processes
Williams, T. P., Chopra, V., and Chikanya, S. R. 2018. [27]	“It isn’t that we’re prostitutes”: Child protection and sexual exploitation of adolescent girls within and beyond refugee camps in Rwanda	(1) Stigma negatively played a role for girls who wished to receive services (as seen even when rape occurs). (2) Young people felt deterred from seeking support because they did not have confidence in the support systems that were available to them. There were concerns that accusations of rape would not be taken seriously and that there was a lack of justice. (3) Economic stressors threatened the viability of families. (4) Girls had material needs but few options to meet those needs within the camps. Their families expected them to do domestic work at home. (5) Children reportedly travelled to nearby towns to exchange sex for money or other goods leaving them exposed to HIV, unplanned pregnancies, and STDs
